# A Warning to Fathers, Teachers, and Young Men, in Relation to a Frightful Cause of Insanity, Etc.

**Published:** 1861-07

**Authors:** 


ART. VI.—*A Warning to Fathers, Teachers, and Young Men, in Relation to a frightful Cause of Insanity, and other serious Disorders of Youth.* By W. S. CHIPLEY, M.D., late Professor of the Theory and Practice of Medicine in the Medical Department of Transylvania University, Medical Superintendent of the Eastern Lunatic Asylum, Lexington, Kentucky. 18mo., pp. 188.

IN this little work Dr. Chipley purposes to lay before young men the evil consequences resulting from indulgence in what is reputed to be the sin of Onan, and also to point out to parents and teachers the causes and mode of prevention of the vice. In the fulfillment of this task, he gives us an exceedingly interesting volume, in the form of a popular essay, in which, on the one hand, he skillfully avoids cloaking his meaning in elaborate technical phrases, and at the same time certainly does not shock the modest sensibilities of the public on the other. The position of Dr. Chipley, as superintendent of the Eastern Lunatic Asylum of Kentucky, renders him a peculiarly fit person to give advice in regard to a disease—

for so we may call it—that is filling our lunatic asylums and prisons, and is gaining strength day by day with every step that our boasted civilization makes, until it becomes a matter of serious import to inquire, has modern progress anything to do with the development of abuses of the genital organs? In antiquity there seems to have been no mention made of masturbation, or any of its serious consequences. There is no mention of it in the Bible; although the vice has Onan for its godfather, there is no proof that he was a masturbator. In compliance with the customs of the ancient Hebrews, he was obliged to marry the widow of his elder brother who had died childless. If he were to have children by her, the first-born would bear the name and inherit the estate of the deceased, "in order that his name might not be blotted out from Israel." Onan, to escape the disgrace which would have resulted from his refusal to marry the widow, did so; but not wishing to injure his own interest or those of his existing children, by having offspring from such a marriage, after he had effected penetration, spilt his semen on the ground. The Hebrew of the original gives reason for such interpretation. The words *im, bo,* are translated "when he had gone into," which certainly means penetration. Lallemand says that every day conscientious husbands do the same thing, either to avoid increasing their family beyond their means, or in consequence of the danger that pregnancy would entail upon their wives, so that the motives of Onan alone were blamable; his act in itself had nothing in common with masturbation. The sacred writings, then, afford no proofs of the prevalence of the vice; nor do the works of the classic poets, who certainly have concealed nothing of the infamous immorality of ancient times, afford us any reason to believe that it was practiced, while the same reticence is observed on the part of the medical writers of those days. During the dark ages, through the feudal era, and, in the midst even of the licentiousness of the *ancien régime,* we hear nothing of it, and we are led to the unflattering conviction that it is essentially a vice of modern times; that where religion, science, and education extend most widely their elevating influence over the globe, that there should that crime, which is the most injurious and degrading that humanity knows, be most rampant.

The causes, then, of the vice must exist with us, and must be exposed and counteracted, if we wish to extirpate it; and this Dr. Chipley does, as far as the compass of his modest volume allows him. He divides his work into three chapters: on the causes of masturbation, on its consequences, and on its prevention. The chapter on the causes is similar to the chapter on the same subject in Lallemand's elaborate work; and very clearly points out to those having charge of the young the numerous apparently trivial circumstances which lead to the implanting of the fatal habit. Late rising, feather beds, imprudent or vicious acts of the attendants of children, disease of the bladder, an elongated prepuce, ascarides in the rectum, neglect of bodily exercises, the assembling of boys in the dormitories of schools, etc., are quoted as inducing circumstances. The author, in remarking on the irritation of the glans penis by sebaceous matter contained under the prepuce, asserts that it is a common cause, the friction used to allay the itching exciting in the mind of the youth the idea of the habit, and hence considers the performance of the Mosaic rite as necessary in a sanitary point of view, and adduces in his support the authority of Dr. Copland, who says: "The neglect of circumcision in Christian countries is certainly no mean physical cause of the prevalence of this vice, and of many of the consequences which follow."

The chapter on the effects of masturbation presents a horrid catalogue of evils of body and mind. All persons do not suffer alike: in some the mind is impaired, in others the body; none escape with impunity. Deformity may result from arrest of development. Dyspepsia, necessitating a stimulant regimen, ending in drunkenness, occurs. All the principal organs of the body participate,—lungs, liver, heart, and brain. What physician is there who has not been consulted for disease of the heart, which a moment's examination has convinced him to be a functional disorder, the result of self-abuse? while chorea and epilepsy, with affection of all the senses, are frequent. Insanity is quoted as one of the most frequent and destructive consequences, and some interesting statistics on the subject are given; and though, in one case—that of the Massachusetts Lunatic Asylum—the percentage of those who had become insane through masturbation is as high as 32 in 100, yet Dr. Chipley believes that truth would justify a still higher scale, affirming, with Guislain, that, where mental alienation comes on without manifest reason, the cause, in young people, should be looked for in Onanism. Impotency is a common sequence, and frequently leads to suicide.

The prevention and cure of the vice occupy the last chapter. Muscular exercise, early rising, and separation of the sexes at an early age, are insisted upon as preventives; while, for those who have already contracted the habit, the author recommends that they be made cognizant of its fatal results, and submit themselves to a proper course of treatment. He gives no detailed plan of cure, as he believes that this would be out of place in a popular work. Altogether, it is a well-written little volume, presenting nothing absolutely novel to the professional reader, but eminently well adapted for the purpose for which it is designed, viz., of awakening a proper feeling in the minds of parents as to the magnitude of the vice, and of pointing out lucidly and honestly its means of detection and prevention; and its dissemination in the hands of parents and teachers would result in an amount of good that must amply repay its philanthropic author.

